# Non-invasive predictors of axillary lymph node burden in breast cancer: a single-institution retrospective analysis

**DOI:** 10.1007/s10549-022-06672-7

**Published:** 2022-07-21

**Authors:** Victoria Ngai, Justina Cheh Juan Tai, Saima Taj, Heba Khanfar, Elefterios Sfakianakis, Athanasios Bakalis, Rose Baker, Muneer Ahmed

**Affiliations:** 1 University College London Medical School, London, UK; 2 Division of Surgery and Interventional Science, University College London, London, UK; 3 Department of Breast Surgery, Royal Free London NHS Foundation Trust, London, UK; 4 Department of Statistics, School of Business, University of Salford, Salford, UK

**Keywords:** Breast cancer, Axillary staging, Axillary ultrasound, Sentinel lymph node biopsy, Axillary lymph node dissection

## Abstract

**Purpose:**

Axillary staging is an important prognostic factor in breast cancer. Sentinel lymph node biopsy (SNB) is currently used to stage patients who are clinically and radiologically node-negative. Since the establishment that axillary node clearance (ANC) does not improve overall survival in breast-conserving surgery for patients with low-risk biological cancers, axillary management has become increasingly conservative. This study aims to identify and assess the clinical predictive value of variables that could play a role in the quantification of axillary burden, including the accuracy of quantifying abnormal axillary nodes on ultrasound.

**Methods:**

A retrospective analysis was conducted of hospital data for female breast cancer patients receiving an ANC at our centre between January 2018 and January 2020. The reference standard for axillary burden was surgical histology following SNB and ANC, allowing categorisation of the patients under ‘low axillary burden’ (2 or fewer pathological macrometastases) or ‘high axillary burden’ (> 2). After exploratory univariate analysis, multivariate logistic regression was conducted to determine relationships between the outcome category and candidate predictor variables: patient age at diagnosis, tumour focality, tumour size on ultrasound and number of abnormal lymph nodes on axillary ultrasound.

**Results:**

One hundred and thirty-five patients were included in the analysis. Logistic regression showed that the number of abnormal lymph nodes on axillary ultrasound was the strongest predictor of axillary burden and statistically significant (*P* = 0.044), with a sensitivity of 66.7% and specificity of 86.8% (*P* = 0.011).

**Conclusion:**

Identifying the number of abnormal lymph nodes on preoperative ultrasound can help to quantify axillary nodal burden and identify patients with high axillary burden, and should be documented as standard in axillary ultrasound reports of patients with breast cancer.

## Introduction

Axillary lymph node status is an important prognostic factor in breast cancer, given its role in defining the pathological stage of breast cancer. Surgical management of the axilla classically initially involves either axillary node clearance (ANC, also known as axillary dissection), where all axillary lymph nodes are removed, or sentinel node biopsy (SNB), where only the first nodes draining the tumour are located and excised for analysis to inform further management. While ANCs were performed liberally in the past, management of the axilla has evolved to become more conservative. This is in light of research identifying subsets of patients—usually those with low-risk cancers who clinically and radiologically show no sign of axillary metastases—for whom upfront ANC is not justified given the lack of clear benefit and risk of lymphoedema, stiffness, neuropathy and infection [1–4]. For these patients, SNBs are first performed in the hope of identifying patients with low axillary burden, who may be spared ANC without any difference in survival outcome.

This move towards the minimization of unnecessary invasive interventions has been received widely. However, there is emerging literature questioning the justification for performing SNBs on *all* patients who do not receive an ANC, as SNB itself, though more conservative than ANC, is still an invasive procedure that is associated with its own risks of post-operative morbidity [5, 6]. Moreover, it has been reported that about 70% of SNBs return negative, so attention has recently turned towards the identification of new tools that could help further stratify patients who would truly benefit from SNB based on their risk of lymph node metastasis [7, 8]. Accurate preoperative quantification of axillary burden would help to better select patients to proceed with ANC, SNB, or, to omit upfront surgical management of the axilla altogether, avoiding overtreatment of the axilla and unnecessary post-operative complications.

Non-invasive assessment of the axilla comprises clinical examination (palpation of the tumour/lymph nodes) and radiological examination (MRI, CT, ultrasonography) [8–11]. Ultrasonography allows for direct visualisation of the axilla and can be used by the breast surgeon in the clinic. Its utility for informing the management of early breast cancer patients has been the subject of recent studies, including the SOUND (Sentinel node vs. Observation after axillary Ultra-souND) study, an ongoing multicentre randomised controlled trial (RCT) based in Italy that is due for completion this year [12, 13]. Another large-scale RCT that was more recently initiated in Germany and Austria, the INSEMA (Intergroup-Sentinel-Mamma) trial, investigates the hypothesis that oncological outcomes are comparable in node-negative breast cancer patients who receive SNB compared to similar patients who receive no axillary surgery [14]. In the context of the possibility that SNB could be avoided in some patients, the value of determining axillary burden with alternative methods becomes apparent.

Similarly, a more reliable, validated way of assessing axillary node burden non-invasively may also better identify patients with a high axillary burden who should receive ANC upfront without the need for a preceding SNB—this, too, would aid in avoiding unnecessary axillary surgery. Studies investigating the diagnostic accuracy of axillary ultrasound alone for determining axillary node involvement have yielded variable sensitivities and specificities [15–18]. Furthermore, the use of axillary ultrasound in combination with other non-invasive clinical or radiological parameters has been less studied and should not be ruled out prematurely.

We therefore aim to examine axillary ultrasound alongside other clinicopathologic variables to assess the quantification of axillary burden, through retrospective review of patients at our hospital for whom axillary status is known from both SNB and ANC. Previous studies have compared axillary ultrasound findings to SNB, but few have included findings from ANC in the analysis. By studying a group of patients who have all received ANC, this study aims to identify patients who could potentially avoid unnecessary overtreatment of the axilla if non-invasive predictors of axillary burden are taken into account.

## Methods

### Study population and data collection

We retrospectively identified breast cancer patients who received primary surgery and underwent ANC between January 2018 and January 2020 at a large teaching hospital in London, United Kingdom. Electronic medical records were reviewed for data collection. The following data were collected: patient age at diagnosis, molecular subtype, tumour focality, palpability of tumour(s), palpability of lymph node(s), tumour size on ultrasound, tumour size on mammogram, number of abnormal lymph nodes found on ultrasound, axillary procedures performed, and number of pathological macrometastases on surgical pathology reports. Molecular subtype was determined by oestrogen receptor (ER), progesterone receptor (PR), and human epidermal growth factor receptor 2 (HER2) status. Patients who did not undergo ANC were excluded so that the total number of pathological macrometastases could be ascertained for every patient. This study was registered locally in accordance with local research and development guidelines.

### Imaging data

Preoperative axillary ultrasound images and reports were reviewed to identify the number of abnormal lymph nodes seen on imaging. ‘Abnormal’ lymph nodes were defined as lymph nodes with an irregular shape, thickened cortex with a diameter of > 3 mm, or absent hilum. Tumour sizes were ascertained from ultrasound and mammogram reports based on the largest tumour diameter provided. Tumour focality (unifocal or multifocal) was determined from mammogram reports.

### Surgical treatment and pathological evaluation

Surgical management of the axilla was performed based on guidelines published by the National Institute of Health and Care Excellence in the United Kingdom and the outcomes of multidisciplinary team meetings at our hospital [19]. SNB was performed for patients who were node-negative in the axilla on palpation, axillary ultrasound or ultrasound-guided needle biopsy. Of these patients, those who were found to have 1 or more sentinel lymph node macrometastases from pathological processing of SNB samples received a subsequent ANC. Patients who were preoperatively found to be node-positive from ultrasound-guided needle biopsy received ANC upfront. SNB was uniformly performed using the dual tracer technique with blue dye and a hand-held gamma probe. Nodes retrieved during SNB and ANC were sent to the pathology department in the same hospital for histological assessment. The presence of isolated tumour cells, micrometastases, macrometastases or no metastasis in the axilla was reported by Consultant Pathologists.

The gold standard to determine true axillary burden was post-surgical pathological analysis as described above. The number of macrometastases identified from SNB and ANC was summed for patients who underwent both procedures, while the number of macrometastases from the ANC alone was taken for patients who did not undergo SNB. For data analysis, true axillary burden was categorised as ‘low’ if there were 2 or fewer positive nodes, or ‘high’ if there were more than 2 positive nodes, based on a differentiation between degrees of axillary burden proposed by the American Society of Clinical Oncologists following the ACOSOG Z0011 trial [20, 21].

### Statistical analysis

All analyses were conducted using the statistics package MINITAB. An available-case analysis approach was taken with respect to missing data. Univariate analysis was first conducted to explore associations between the identified variables and low/high axillary burden. A Welch’s *t* test was performed for continuous variables (age at diagnosis, tumour size on ultrasound and tumour size on mammogram). A chi-squared test was performed for categorical variables (tumour focality, tumour palpability, lymph node palpability, lymph node burden on ultrasound). From the univariate analysis, variables showing a correlation with true axillary burden at a significance of *P* < 0.15 were included for multivariate analysis. Multivariate logistic regression was performed on these variables to identify potential predictors of axillary burden, and *P* < 0.05 was considered to be statistically significant for the final result.

Clinical performance metrics were calculated for any variables that were identified from logistic regression analysis to be statistically significant predictors of true axillary burden, namely the positive predictive value, negative predictive value, sensitivity, specificity and accuracy (defined as the proportion of true positive and true negative cases summed out of all cases analysed). False negative, false positive and correctly predicted cases were stratified by disease characteristics—namely histological subtype, molecular subtype and grade—to identify any variability in the performance of statistically significant predictors amongst these disease subtypes. All figures were produced in Microsoft® Excel and Microsoft® Word.

## Results

### Patient characteristics

**Fig. 1:**
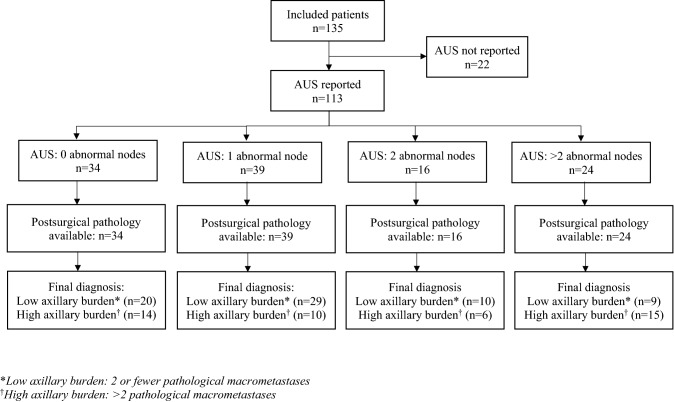
Axillary burden quantification from axillary ultrasound compared to gold standard pathological analysis

Of the 135 patients included, the median age at diagnosis was 57 years (age range 26–89 years). All included patients were female. Table 1 shows the distribution of breast cancer molecular subtypes among the patients, the axillary procedures received and category of axillary burden. A total of 51 cases of high axillary burden (> 2 pathological macrometastases) and 84 cases of low axillary burden (2 or fewer pathological macrometastases) were identified based on pathological reports. Figure 1 shows the distribution of numbers of abnormal nodes found on axillary ultrasound, compared to the axillary burden based on gold standard post-surgical pathology.

**Table 1:** Patient characteristics [*n* = 135]

	Number of patients	(%)
Histological subtype		
Ductal	114	84
Lobular	13	10
Other types	6	4
Molecular subtype		
Luminal A (ER-positive and PR-positive)	84	62
Luminal B (ER-positive and PR-negative)	19	14
HER2-positive (luminal and non-luminal)	43	32
Triple-negative	19	14
Axillary procedure		
Sentinel lymph node biopsy (SNB)	39	29
Axillary node clearance (ANC)	135	100
Axillary burden		
2 or fewer pathological macrometastases	51	38
> 2 pathological macrometastases	84	62

### Univariate analysis

The details of univariate analysis are shown in Tables 2 and 3. Age at diagnosis, tumour focality, tumour size on ultrasound and number of abnormal lymph nodes (LNs) found on axillary ultrasound showed an association with true axillary burden at *P* < 0.15. Of these variables, age at diagnosis and number of abnormal lymph nodes found on axillary ultrasound were significant at *P* < 0.05. Palpability of the tumour or lymph nodes upon clinical examination and the tumour size on mammogram did not show a statistically significant correlation with true axillary burden.

**Table 2:** Results of chi-squared univariate analysis for categorical variables

	*n* (low axillary burden)^a^	*n* (high axillary burden)^b^	χ^2^	*P* value
Tumour focality			2.64	0.105
Unifocal	77	42		
Multifocal	7	9		
Clinical examination: tumour			0.201	0.634
Palpable tumour	72	44		
Non-palpable tumour	9	7		
Clinical examination: lymph nodes			1.49	0.223
Palpable lymph nodes	17	16		
Non-palpable lymph nodes	61	35		
Number of abnormal LNs on ultrasound (binary groups)			6.14	0.011
2 or fewer	59	30		
> 2	9	15		
Number of abnormal LNs on ultrasound			8.49	0.037
0	20	14		
1	29	10		
2	10	6		
> 2	9	15		

^a^Low axillary burden: 2 or fewer pathological macrometastases

^b^High axillary burden: > 2 pathological macrometastases

**Table 3:** Results of Welch’s *t* test univariate analysis for continuous variables

	Mean		*P* value
	Low axillary burden^a^	High axillary burden^b^	
Age at diagnosis (years)	56.8	61.8	0.032
Tumour size on ultrasound (mm)	13.9	17.7	0.082
Tumour size on mammogram (mm)	31.7	35.7	0.316

^a^Low axillary burden: 2 or fewer pathological macrometastases

^b^High axillary burden: > 2 pathological macrometastases

### Multivariate analysis

The coding of raw data for the number of abnormal lymph nodes on ultrasound did not allow for its analysis as a continuous variable, so for the purposes of logistic regression analysis, this variable is presented as a binary variable of ‘2 or fewer’ versus ‘ > 2’ abnormal nodes. This categorisation aligns with our differentiation between low and high axillary burden based on the number of pathological macrometastases identified from axillary surgery.

Multivariate logistic regression showed that the number of abnormal lymph nodes found on axillary ultrasound was a significant predictor of true axillary burden with an odds ratio (OR) of 2.82 (95% CI 1.03–7.72, *P* = 0.044). Age at diagnosis (OR 1.02, *P* = 0.177), tumour focality (OR 2.38, *P* = 0.128) and tumour size on ultrasound (OR 1.01, *P* = 0.382) showed an odds ratio of > 1 but did not reach *P* < 0.05, and thus were not considered statistically significant. The results of logistic regression analysis are shown in Table 4.

**Table 4:** Results of multivariate logistic regression

	Odds ratio	95% Confidence interval		*P* value
		Lower	Upper	
Age at diagnosis (years)	1.02	0.99	1.05	0.177
Tumour focality (unifocal versus multifocal)	2.38	0.78	7.27	0.128
Tumour size on ultrasound (mm)	1.01	0.99	1.04	0.382
Number of abnormal LNs on ultrasound (2 or fewer versus > 2 abnormal LNs)	2.82	1.03	7.72	0.044

### Performance of axillary burden on ultrasound as a predictor

**Fig. 2:**
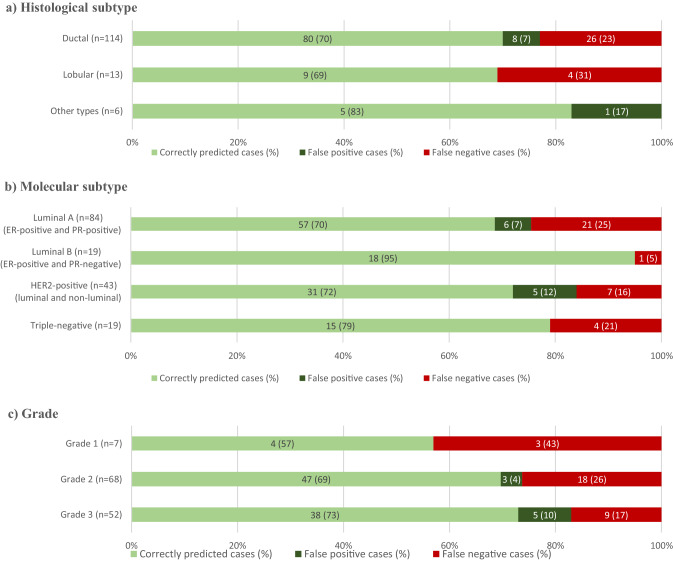
Outcome of axillary ultrasound as a predictor of high axillary burden (> 2 pathological macrometastases) for different tumour types

Table 5 shows that axillary burden on ultrasound (2 or fewer abnormal nodes versus > 2 abnormal nodes) had a sensitivity of 33%, specificity of 87%, positive predictive value of 63% and negative predictive value of 66%. The accuracy of this variable overall as a predictor of axillary burden was 65%. Figure 2 shows the variability in performance of this variable as a predictor in patients with different tumour types.

**Table 5:** Clinical performance metrics of axillary burden on ultrasound as a predictor of true high axillary burden (> 2 pathological macrometastases)

	True axillary burden			
	2 or fewer pathological macrometastases	> 2 pathological macrometastases	Total	
Axillary burden on ultrasound				
2 or fewer abnormal nodes	59	30	89	Negative predictive value = 66%
> 2 abnormal nodes	9	15	24	Positive predictive value = 63%
Total	68	45		Accuracy = 65%
	Specificity = 87%	Sensitivity = 33%

## Discussion

The results of both univariate and multivariate analyses show that the number of abnormal lymph nodes found on axillary ultrasound is a statistically significant predictor of axillary burden, while data obtained from clinical examination (tumour and lymph node palpability) showed non-significant correlations with axillary burden. This corroborates the findings of a recent review by Marino et al. of diagnostic tools in lymph node assessment for primary breast cancer patients, which concluded that ultrasound was the method of choice worldwide for assessing lymph node involvement, even though mammography is the standard for breast screening [22]. This review also concluded that physical examination has a low accuracy in predicting nodal burden, which was corroborated by our results.

In previous studies on lymph node assessment using ultrasound for early breast cancer patients, > 2 or > 3 abnormal lymph nodes on ultrasound were found to predict high axillary burden. The authors indicated that these parameters could help to select patients who may proceed to ANC without the need for SNB [4, 23–25]. These findings reinforce the utility of preoperatively determining the number of abnormal lymph nodes on ultrasound. One such study was that by Yi et al., who included 347 patients with suspicious findings on a mammogram and concluded that ≥ 3 abnormal lymph nodes on axillary ultrasound, alongside a non-circumscribed tumour margin and cortex thickness ≥ 3 mm, were factors that best predicted high axillary burden with a high sensitivity and negative predictive value [26]. However, one of the limitations mentioned by the authors in their study was that not all patients received an ANC, so the axillary burden of node-negative patients could not be verified. In our study where all included patients received an ANC, we found that out of all the patients with zero abnormal lymph nodes on ultrasound, 14 (41.2%) went on to show a high axillary burden on pathological analysis. Despite this, our statistical analyses were still concordant with the findings of Yi et al. in that the number of nodes found on ultrasound significantly predicted axillary burden.

In our study, we found that the specificity of the number of abnormal nodes on ultrasound was higher than the sensitivity, positive predictive value or negative predictive value. This suggests that axillary ultrasound may be a useful tool in avoiding unnecessary SNB and associated post-operative morbidity, in patients with high axillary burden who could receive ANC upfront. However, the positive and negative predictive value of below 70% suggest that axillary ultrasound alone may not be sufficient to replace SNB. We also observed a variability in performance amongst subtypes of breast cancer. Increased underestimation of axillary burden in invasive lobular carcinoma has been previously reported and was also observed in our sample, suggesting that ultrasound alone may not be suitable to stage the axilla in all patients [27]. This relationship did not reach statistical significance with the current sample size but is worthy of study in larger cohorts.

The limitations of using axillary ultrasound alone to investigate axillary burden could be overcome by considering ultrasound alongside other investigations to inform management of the axilla, such as fine-needle aspiration cytology which has been shown to improve the positive and negative predictive value [28]. Other imaging modalities can also be used in cases where ultrasound is found to be less accurate. For example, in patients with invasive lobular carcinoma, MRI has been found to predict high axillary burden [27].

With regard to tumour size, a number of studies have found this parameter to be predictive of the presence of lymph node metastasis (versus no lymph node involvement at all) [29–31]. The studies were not able to determine whether this relationship prevailed when it came to distinguishing between patients with high versus low lymph node burden. The results of our univariate analysis show that the tumour size on ultrasound could play a role in predicting high/low axillary burden. However, multivariate analysis showed that the significance of this parameter was not as apparent as it had been in other studies. This discrepancy suggests that directly examining the lymph nodes instead of the tumour may aid in predicting axillary lymph node burden more precisely.

Multifocality of the tumour showed a moderate correlation with axillary burden in both statistical tests, but the result was not statistically significant in either test. It is worth noting that previous work has found multifocality to be a predictor of axillary burden [32, 33]. The result in our study could be in part due to a smaller sample size compared to the studies cited above.

There are a few limitations to this study, including its retrospective nature which restricted data collection to that which was pre-existing in medical records. This single-centre study had a sample size of 135 patients, and thus would benefit from validation in prospective cohorts or larger sample sizes. Any variables involving the use of ultrasound are subject to the proficiency of the user and results can be operator-dependent. In our case, axillary ultrasound scanning was conducted by radiologists in a tertiary hospital setting. The clinical translation of this study’s findings would be dependent on the standardisation and training of clinicians to identify abnormal lymph nodes in the axilla on ultrasound, including non-radiologists such as surgeons if they were to perform axillary ultrasound scanning. Specific ultrasound features, other than number of abnormal nodes, that can distinguish patients with high axillary burden were beyond the scope of this study, but this has been explored in other studies [22, 26, 31].

This study builds on a growing body of research on the axillary assessment of breast cancer patients and the identification of criteria for patients who are more or less likely to benefit from axillary surgery (sentinel node biopsy or axillary clearance). Following the evidence presented above that ultrasound investigation of axillary lymph nodes can help to quantify axillary burden, the exploration of a scoring system for ultrasound assessment in breast cancer patients could also be an area of focus for future research and clinical practice.

## Conclusion

Identifying the number of abnormal lymph nodes on axillary ultrasound can play a role in quantifying axillary nodal burden in breast cancer patients. In particular, it may be useful in identifying patients with a high axillary burden of more than two macrometastases, to consider receiving ANC upfront or primary systemic therapy. However, the accuracy of axillary ultrasound alone is not sufficient to replace SNB for all patients. Further research comparing the role of a wider range of ultrasound features and imaging modalities in staging the axilla could inform future management of the axilla with reduced surgical morbidity.

## Data Availability

The datasets generated during and analysed during the current study are not publicly available due to institutional restrictions but are available from the corresponding author on reasonable request.
